# Bleeding “Dieulafoy’s-like” lesion resembling the duodenal papilla: a case report

**DOI:** 10.1186/s13256-015-0594-8

**Published:** 2015-05-23

**Authors:** Mohammad Bilal, Anastasios Kapetanos, Haider Ali Khan, Shyam Thakkar

**Affiliations:** 1 Allegheny General Hospital, 320 East North Avenue, Pittsburgh, PA 15212 USA; 2 Center for Liver and Digestive Diseases, Holy Family Hospital, Satellite Town, Rawalpindi, 46000 Pakistan

**Keywords:** Dieulafoy’s lesion, Duodenal papilla, Gastrointestinal bleeding

## Abstract

**Introduction:**

Dieulafoy’s lesion is an uncommon but important cause of gastrointestinal bleeding in which hemorrhage occurs from a pinpoint, non-ulcerated arterial lesion. DLs are usually located in the stomach, most commonly in people between the ages of 50 and 70 years. In this report, we describe a teenage patient with an unusual presentation of a bleeding duodenal Dieulafoy’s-like lesion that resembled the duodenal papilla.

**Case presentation:**

An 18-year-old Pakistani woman presented to our emergency department with hematemesis of 6 hours’ duration. Her past medical history was unremarkable. A nasogastric aspirate was negative for blood. The patient’s hemoglobin was found to be 4g/dl. She was resuscitated with intravenous fluids and blood transfusion. An esophagogastroduodenoscopy was performed, which revealed swelling in the first part of the duodenum, the initial appearance of which suggested that it was an abnormally placed or accessory papilla. There was a small, <3–mm opening on the lesion that resembled the biliary or pancreatic orifice. On gentle manipulation with a catheter, blood spurted from the swelling area, and a vessel was visible. Adrenaline was used for hemostasis. After hemostasis was achieved, it became clear that the lesion was most consistent with a Dieulafoy’s-like lesion and not a papilla. Band ligation was then performed, and the patient did not develop any complications and did not have any further episodes of bleeding. The patient was eventually discharged to home in stable condition.

**Conclusions:**

This case report highlights the importance of considering a DL as a cause of small-bowel hemorrhage and recognizing its potential resemblance to the papilla. Although the endoscopic diagnostic criteria for a Dieulafoy’s lesion have been described in great detail, there is a paucity of literature describing a Dieulafoy’s lesion or a similar lesion resembling the duodenal papilla.

## Introduction

Dieulafoy’s lesion (DL) is an uncommon but important cause of gastrointestinal (GI) bleeding. Although the great majority of DLs are located in the stomach within 6cm of the gastroesophageal junction, DLs in other parts of the GI tract are rare. DLs rarely are the cause of small-bowel hemorrhage. They are rarely seen in teenagers. In this report, we describe a teenage patient with an unusual presentation of a bleeding duodenal Dieulafoy’s-like lesion that resembled the duodenal papilla.

## Case presentation

**Figure 1:**
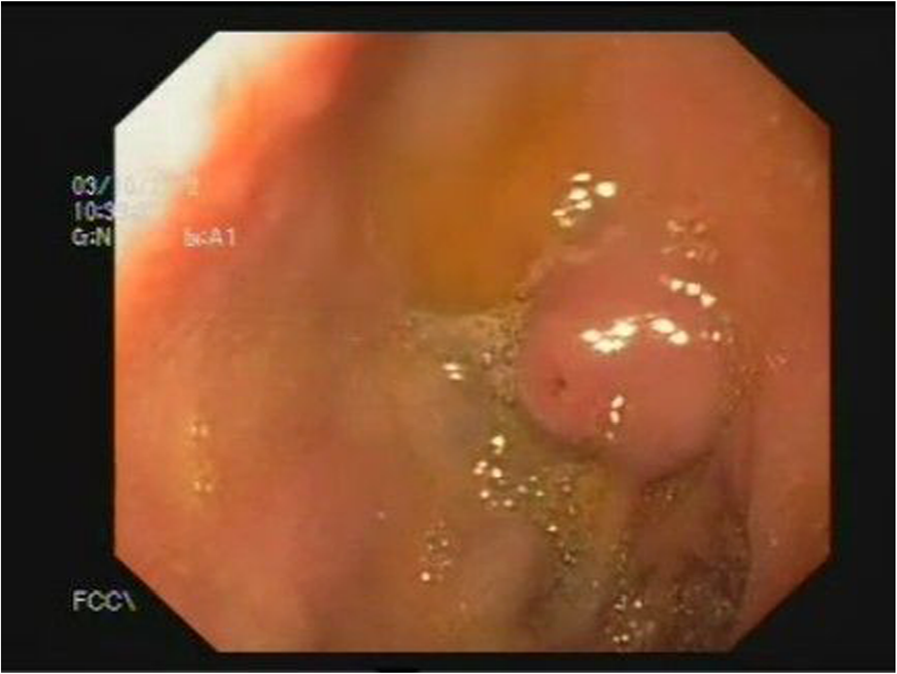
Dieulafoy’s-like lesion resembling the duodenal papilla seen during upper endoscopy. The lesion was a small, <3-mm mucosal defect with normal surrounding mucosa.

**Figure 2:**
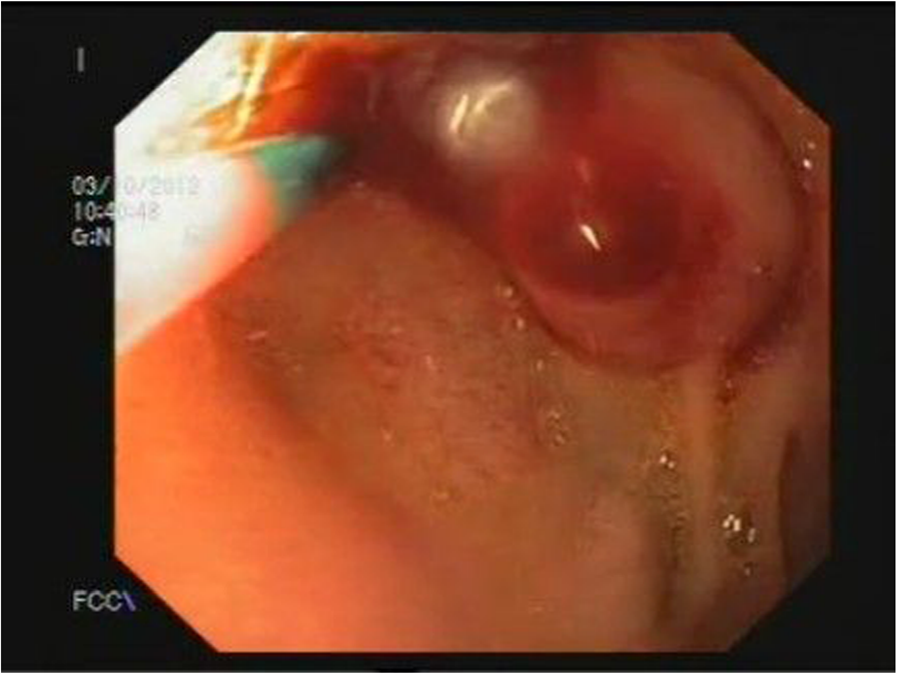
Bleeding Dieulafoy’s-like lesion after gentle manipulation and visualization of the bleeding vessel seen during upper endoscopy.

**Figure 3:**
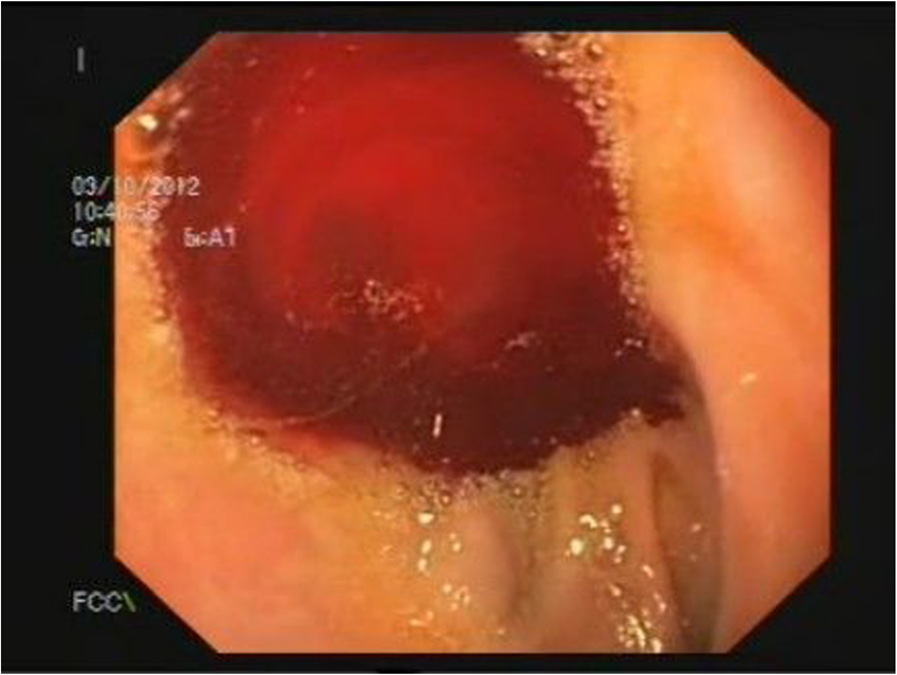
Dieulafoy’s-like lesion leading to massive bleeding seen during upper endoscopy.

**Figure 4:**
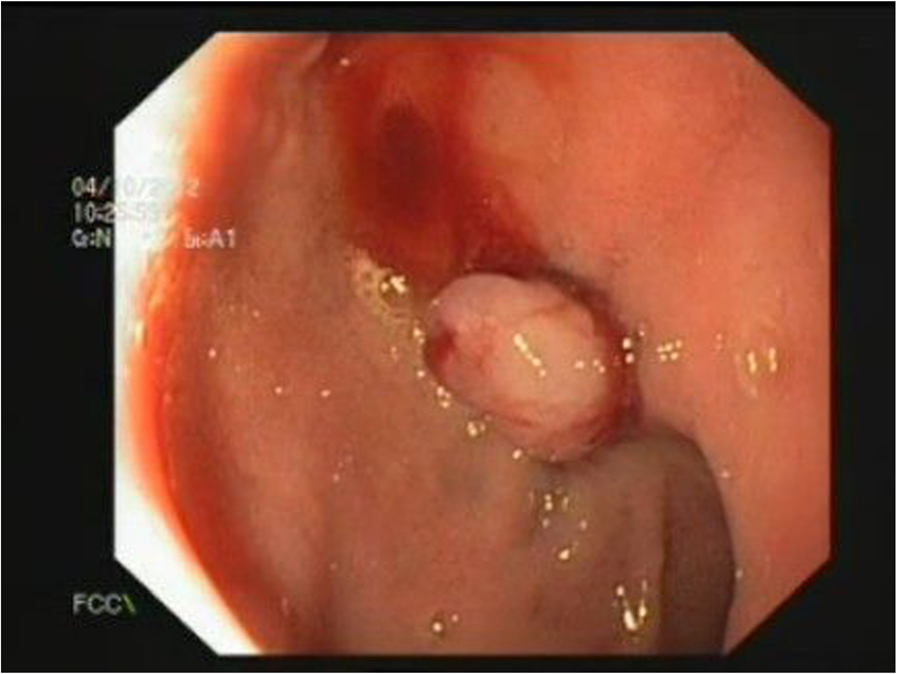
Dieulafoy’s-like lesion after band ligation seen during upper endoscopy.

An 18-year-old Pakistani woman presented to our emergency department with hematemesis of 6 hours’ duration. She did not have any past medical history of GI bleeding, chronic liver disease, peptic ulcer disease or use of alcohol or non-steroidal anti-inflammatory drugs. She was taking no medications prior to this presentation and denied use of any herbal or dietary supplements. Her family history was significant for hypertension in her father. Her family had no history of liver disease, colon cancer or any other GI malignancy. Her physical examination was significant for tachycardia and lethargy. Her pulse rate was 122 beats/min, and her blood pressure was 100/60mmHg. Her respiratory rate was 18 breaths/min, and her body temperature was 37.6°C. She had no abdominal pain or any stigmata of chronic liver disease. A nasogastric aspirate was negative for blood. Her laboratory values were as follows: hemoglobin, 4g/dl (normal range, 12.3 to 15.3g/dl); mean corpuscular volume, 77.2 fl (normal range, 80.0 to 96.0 fl); white blood cell count, 9000/mm^3^ (normal range, 4.8 to 10.8×10^4^ mm^3^); platelets, 238,000/μl (normal range, 145,000 to 445,000/μl); urea, 26mg/dl (normal range, 5 to 22mg/dl); creatinine, 0.8mg/dl (normal range, 0.6 to 1.3mg/dl); serum sodium, 136mmol/L (normal range, 134 to 142mmol/L); serum potassium, 3.2mmol/L (normal range, 3.5 to 5.0mmol/L); total bilirubin, 0.6mg/dl (normal range, 0.2 to 1.2mg/dl); aspartate transaminase, 32IU/L (normal range, 5 to 34IU/L); alanine transaminase, 40IU/L (normal range, 5 to 55 IU/L); alkaline phosphatase, 87IU/L (normal range, 42 to 140IU/L); international normalized ratio, 0.9 (normal range, 0.9 to 1.1); and prothrombin time, 12.9 seconds (normal range, 11.8 to 14.3 seconds). She was resuscitated with intravenous fluids and 4U of packed red blood cells. An esophagogastroduodenoscopy was performed, which revealed swelling in the first part of the duodenum (Figure 1). The initial appearance of the swelling suggested that it was an abnormally placed or accessory papilla. There was a small, <3-mm opening on the lesion that resembled the biliary or pancreatic orifice. On gentle manipulation with a catheter, blood spurted from the swelling area, and a vessel was visible (Figures 2 and 3). Adrenaline was used for hemostasis. After hemostasis was achieved, it became clear that the lesion was most consistent with a Dieulafoy’s-like lesion and not a papilla. Band ligation was then performed (Figure 4), and the patient did not develop any complications and did not have any further episodes of bleeding. The patient was eventually discharged to home in stable condition. The patient was followed up for 6 months and did not have any other episodes of GI bleeding.

## Discussion

DL is an uncommon but important cause of GI bleeding in which hemorrhage occurs from a pinpoint, non-ulcerated arterial lesion [1,2]. DLs have the greatest propensity to develop in the stomach, within 6cm of the gastro-esophageal junction [3].

A number of features make our patient’s Dieulafoy’s-like lesion presentation noteworthy. DLs rarely present as small-bowel hemorrhage [4,5]. DLs that resemble the papilla make endoscopic detection challenging, are uncommonly recognized in teenagers and are mostly commonly seen in patients between 50 and 70 years of age [6].

The endoscopic criteria for diagnosing DLs include any one of the following three abnormalities: (1) active arterial spurting or micropulsatile streaming from a mucosal defect <3mm or through normal surrounding mucosa; (2) visualization of a protruding vessel with or without bleeding, within a minute mucosal defect or through normal surrounding mucosa; and/or (3) the appearance of a fresh, densely adherent clot with a narrow point of attachment to a minute mucosal defect [7]. Our patient met two of the three criteria for diagnosing a DL endoscopically. Specifically, she had a mucosal defect <3mm in size with active bleeding surrounding normal mucosa (Figure 1). Additionally, a vessel was visualized once bleeding started (Figure 2). Differential diagnostic possibilities considered included hemosuccus pancreaticus or hemobilia, if the lesion was an abnormally placed papilla. However, the patient did not develop jaundice or pancreatitis after band ligation of the lesion, thus making these entities less likely. One other differential diagnostic consideration would be bleeding from a submucosal tumor. However, endoscopic manipulation demonstrated a soft lesion inconsistent with a GI stromal tumor or carcinoid tumor. A bleeding submucosal lipoma was a possibility, as lipomas are soft; however, the lesion lacked the characteristic yellow hue seen in lipomas. As such, the endoscopic findings were most consistent with a lesion similar to a DL. The lesion was thus identified as a Dieulafoy’s-like lesion. In our patient, the appearance of the lesion mimicked an accessory or abnormally placed papilla. When considering therapy for such lesions, it is important to ensure that the lesion is not the actual papilla, because accidental band ligation of the papilla can lead to complications such as acute pancreatitis and obstructive jaundice.

Present-day options for treatment of DL include endoscopic therapy in the form of endoscopic clipping or dual therapy of four-quadrant diluted epinephrine injection (1:10,000) with thermal coaptive coagulation [7].

Although there is no consensus regarding treatment of DL, endoscopic treatment has evolved over the years as the first-line therapy for DL [8]. Endoscopic treatment has been further classified into regional injections, mechanical therapy and thermal therapy [9].

Regional injection therapy consists mainly of local epinephrine injections and sclerotherapy. Studies have shown that use of epinephrine alone in management of DLs has a higher incidence of rebleeding [10]. Widely used thermal therapies include argon plasma coagulation (APC), electrocoagulation and heat probe coagulation. In one single-center study, researchers reported that use of APC alone is an effective way of managing DLs and that there is less chance of rebleeding [11]. Mechanical therapy consists of band ligation and hemostatic clips placement. Studies have shown mechanical therapy to be superior to injection therapy for managing DLs, with a significantly lower rebleeding rate with mechanical therapy [12]. Several studies and case reports have shown band ligation to be an effective modality for treatment of DLs in different locations of the GI tract [13-15]. One randomized control trial showed no difference in the efficacy or safety of the use of band ligation versus hemoclip placement for management of bleeding DLs [8]. Failed attempts at endoscopic therapy include interventional radiology bleed localization and embolization or, rarely, surgery with oversew techniques.

## Conclusions

Although the endoscopic diagnostic criteria for DL have been described in great detail, there is a paucity of literature describing a DL resembling the duodenal papilla. This atypical presentation of a Dieulafoy’s-like lesion, especially in the duodenum, should be borne in mind by endoscopists when searching for a source of bleeding.

## Consent

Written informed consent was obtained from the patient for publication of this case report and any accompanying images. A copy of the written consent is available for review by the Editor-in-Chief of this journal.

## Abbreviations

APC: Argon plasma coagulation
DL: Dieulafoy’s lesion
GI: Gastrointestinal
